# On the Design of Efficient Hierarchic Architecture for Software Defined Vehicular Networks

**DOI:** 10.3390/s21041400

**Published:** 2021-02-17

**Authors:** Muhammad Adnan, Jawaid Iqbal, Abdul Waheed, Noor Ul Amin, Mahdi Zareei, Shidrokh Goudarzi, Asif Umer

**Affiliations:** 1 Department of Information Technology, Hazara University Mansehra, Mansehra 21120, Pakistan; adnan25408@gmail.com (M.A.); jawaid5825@gmail.com (J.I.); namin@hu.edu.pk (N.U.A.); asifumer@hu.edu.pk (A.U.); 2 Department of Computer Science, Capital University of Science and Technology, Islamabad 44000, Pakistan; 3 School of Electrical and Computer Engineering, Seoul National University, Seoul 08826, Korea; 4 Tecnologico de Monterrey, School of Engineering and Sciences, Zapopan 45201, Mexico; m.zareei@tec.mx; 5 Centre for Artificial Intelligent (CAIT), Universiti Kebangsaan Malaysia, Bangi 43600, Malaysia

**Keywords:** VANETs, QoS, SDVN, traffic management, scheduling

## Abstract

Modern vehicles are equipped with various sensors, onboard units, and devices such as Application Unit (AU) that support routing and communication. In VANETs, traffic management and Quality of Service (QoS) are the main research dimensions to be considered while designing VANETs architectures. To cope with the issues of QoS faced by the VANETs, we design an efficient SDN-based architecture where we focus on the QoS of VANETs. In this paper, QoS is achieved by a priority-based scheduling algorithm in which we prioritize traffic flow messages in the safety queue and non-safety queue. In the safety queue, the messages are prioritized based on deadline and size using the New Deadline and Size of data method (NDS) with constrained location and deadline. In contrast, the non-safety queue is prioritized based on First Come First Serve (FCFS) method. For the simulation of our proposed scheduling algorithm, we use a well-known cloud computing framework *CloudSim* toolkit. The simulation results of safety messages show better performance than non-safety messages in terms of execution time.

## 1. Introduction

Recently, VANETs have received great attraction in the research community. The researchers are developing protocols, applications, and simulation tools in different dimensions to make them smarter. In this connection, several architectures were proposed but are still facing some difficulties such as less flexibility, less programmability, less scalability in the deployment of services in large-scale VANETs environment. Similarly, the network throughput problem becomes more sensitive when a large amount of information is simultaneously transferred between the hosts. The situation gets inferior when the network is congested with inefficient routing or bottlenecks. These issues create difficulty in the management of the network due to the dynamic behavior of the VANETs. Therefore, a new networking paradigm was introduced, known as Software Defined Networks (SDN). The basic idea behind SDN is the decoupling of the network control plane from the data plane. The data plane defines forwarding data, while the control plane is responsible for controlling the entire network [1]. The decoupling of the network control plane from the data plane provides a simpler programmable environment and provides external software opportunity to define a network’s behavior.

The integration of SDN and VANETs can play a vital role in developing a new, improved VANETs architecture. With the in-depth study of literature review and comprehensive analysis of these two networking trends (VANETs and SDN), we aim to design a new SDN-based VANETs architecture where the VANETs will be managed in a programmable and centralized way. SDN splits the data plane from the control plane, with centralized network controllers, which conclude how traffic flow will be forwarded within the entire network [2]. We believe that QoS in traffic management is an unavoidable and challenging concern for these two networking trends (VANETs and SDN).

### 1.1. Contributions

The main contributions of this paper are as follows;

1. We proposed a novel efficient architecture for SDVN to improve the QoS using a priority-based scheduling algorithm.
2. We prioritize traffic flow messages both in safety and non-safety queues.
3. In the safety queue, the messages are prioritized based on deadline and size using the NDS with constrained location and deadline.
4. In contrast, the non-safety queue is prioritized based on the FCFS algorithm.
5. We used a well-known cloud computing framework *CloudSim* toolkit to simulate the proposed priority-based scheduling algorithm in hierarchic SDVN architecture.

### 1.2. Paper Organization

The structure of this paper is categorized as follows. Section 2 consists of related work about VANETs and their traffic management, the background of SDN-based VANETs, scheduling schemes used in SDVN and VANETs. Section 3 consists of the proposed scheme. This proposed scheme consists of proposed hierarchic architecture for SDVN that contributes to the QoS in traffic management by proposing a priority-based scheduling algorithm. Section 4 consists of simulation and performance analysis of Priority-based Scheduling Algorithm (PSA) where we used *CloudSim* toolkit to simulate our proposed PSA. Section 5 concludes the paper.

## 2. Related Work

Considering the QoS requirements in SDVN, we move towards an efficient SDVN architecture. For this purpose, a comprehensive literature survey is presented, covering the VANETs, background of SDN, SDN-based VANETs, and QoS factors in traffic management.

### 2.1. Overview of VANETs

Recently, by the rapid development of wireless communication technology and the increased demand in the transportation field’s information technology, the VANETs are an integral element of the Intelligent Transportation System (ITS). VANETs can equip hundreds or thousands of nodes in wireless communication. VANETs is a new type of Ad hoc network and is a particular part of its and is a subclass of Mobile Ad hoc Networks (MANETs) with distinctive properties [3] such as dynamic topology and limited energy. Simultaneously, the VANETs have different characteristics such as mobility, dynamic topology, restricted geographical topology, the density of vehicle that is changeable concerning time, no constraints on network size, road pattern restrictions, etc. VANETs have three communication modes, which are V2V, Vehicles-to-Roadside (V2R) unit, and V2I. VANETs plays an essential role in safety as well as non-safety applications [4]. Driver drowsiness prevention system, emergency warning system, collision avoidance, automatic emergency braking system are included in the safety applications. On the other side, the traffic information systems such as direction changer, cooperative entertainment, toll service, internet access fall under the non-safety applications [3]. Significant applications of VANETs include road information dissemination that helps out to the drivers, provides car safety based on sensor data, accident avoidance, regional weather forecast, information regarding the next available parking space, map location, driverless vehicles, fuel prices offered by the nearest station and many more [4]. To make possible these applications, different protocols have been deployed. The researchers are attracted to developing protocols, applications, and simulation tools in VANETs to improve traffic management efficiency and communication.

### 2.2. Overview of SDN

In [2], Kreutz et al. pointed out that the SDN is superior to traditional networks due to some drawbacks. They do not have global information on the network, manual configuration, and high latency in path recovery. This new networking paradigm SDN is designed with a logical programmable central controller keeping global information. SDN separates the data plane from the control plane with a logically centralized controller and global view of the entire network that decides how traffic flow will be handled within the network. With the *OpenFlow* protocol’s help as southbound Application Programming Interface (API) and northbound API, the control plane’s interaction is accomplished with the data plane and application plane correspondingly.

Mousa et al. [5] design a cost-effective data sharing spectrum depend on CR technology to transmit the vehicle’s information from RSU to infrastructure using 5G to improve the data delivery ratio in VANET. Moreover, centralized control enables rapid reconfiguration of the network, allocating network resources in dynamical ways, is more flexible, and makes troubleshooting more straightforward and more manageable. Goudarzi et al. [6] proposed a new networking paradigm with SDN’s unique properties called Software Defined Networks Edge (SDNE) and categorized the SDNE concept to create taxonomy-based vital characteristics. They identified and outlined the key requirements for SDNE and discussed several challenges that should be addressed to promote SDNE implementation. Moreover, applied the concept of edge computing to overcome the latency and to enhance the performance. A novel three-tier edge computing structure is used to establish the efficient route for vehicle data transmission in VANET.

### 2.3. Overview of SDVN Architecture

Several architectures for SDVN, such as architecture having a central control host, selects server architecture with partial decentralization and hierarchic architecture.

With time, new frameworks are developed to improve existing schemes. In [7], Sadio et al. proposed a topology-based routing protocol using SDN technology. This scheme consists of a routing algorithm through which path is selected, and flow tables are created based on path selection, which is accomplished with the help of the predicted topology. There are two models of communication that are unicast for data collecting and geocast for data dissemination. The performance analysis shows that the SDN is efficient than the other traditional routing protocols. Soufian et al. [8] worked on the architectural elements and placed dynamic controllers in SDVN. The author describes different approaches and proposes an architecture for dynamic controllers to the placement of controllers to readjust the controllers into road traffic situations adoptively. There must be a controller burden due to continuous communication to the forwarding nodes in a centralized SDN controller environment, collecting information about the network state, and applying different forwarding rules and network policies. That is why they proposed dynamic controller architecture for SDVN. The proposed dynamic controller’s strategy is evaluated in a real traffic scenario and shows excellent results to reduce network changes. Soleymani et al. [9] proposed a trust model for VANET based on Multi-access Edge Computing (MEC). This scheme consists of two algorithms: selecting the received information from neighboring in-vehicle messages from V2V and V2I communications. Moreover, the second one is implemented to *OpenFlow* protocol for the updation of flow tables for forwarding devices. This architecture also comprises four logical layers through which it improves the path routing and reduces latency computation. Sadio et al. [10] proposed a prototype to design SDVN. In this scheme, an SDN environment based on the backbone is tested in real hardware that comprises *OpenFlow* switches. The SDN environment based on Radio Access is tested on a Wi-Fi access point comprised of *OpenFlow* switches and sustains click modular router. For better mobility management of V2V and V2I communications, routing algorithms for topology prediction are used on different SDN controllers. As a result, free bandwidth for routing is more suitable because it kept the flow balance through SDN switches.

Al-Mayouf et al. [11] proposed an efficient routing protocol to overcome the transmission delay, packet loss, along with enhancing the efficiency of the VANET for reliable communication of vehicle information from source to destination node. Moreover, the proposed RTISAR algorithm used a novel formula for computing the status of the segment. The proposed scheme results are compared with similar schemes and prove that the RTISAR algorithm is better in terms of packet delivery ratio, transmission delay, and transmission overhead. Thota et al. [12] design a novel protocol to overcome the latency and to improve the reliability among vehicle-to-vehicle communication in a VANET environment. Furthermore, to computes the densities of vehicles in urban and rural areas various technologies are applied and then validate the performance using the application layer.

### 2.4. Scheduling Schemes Used in SDVN and VANET

Software-defined VANETs lack the message priority, and it is essential to send a message on a priority basis. Thus, the authors proposed an algorithm for message prioritization where messages are forwarded based on priority, such as emergency, low, and high priority messages. They implemented the message prioritization inside the *OpenFlow* protocol, which can cause burden and delay. In [13], Ahmed et al. proposed an architecture based on SDN for infrastructure-less VANETs environments known as Unmanned Aerial Vehicle (UAV) assisted. UAVs are integrated to investigate unreachable affected zones and the management of rescue vehicles in case of emergencies. These authors examine a data processing policy that consists of computation offloading/sharing decision problems for better management. The main aim is to keep a balance between energy consumption and delay in terms of computation. A theoretical game approach is used to create offloading/sharing decision problems, and a distributed computation algorithm is designed to solve the problem.

In [14], Smita et al. proposed a scheduling algorithm for VANETs based on a priority-based RSA algorithm (p-RSA) using a dynamic cloud. A dynamic cloud is placed on the roadside unit’s position for maintaining the quality of service to the users. This algorithm divides the services into different categories such as emergency, least, urgent, and average. Hence, the highest priority is given to emergency service among all. The proposed scheduling algorithm is compared with other scheduling algorithms that consist of FCFS, NDS, and Shortest job based on Data First (SDF). They claimed that the proposed scheme algorithms show better results in less bandwidth consumption and less energy use on performing a maximum number of services. In [15], Zhang et al. proposed a scheduling scheme for accessing the data from the vehicle to RSU, based on both deadline and size, known as (D∗S) algorithm. If multiple requests ask for the same deadline among all of these requests in this algorithm, the smallest data size request will serve first. If multiple requests ask for the same data size among all of these requests, the smallest earlier deadline request should be served first. Furthermore, the author enhances the (D∗S) algorithm to (D∗S/N) schedule. Most pending data requests should be provided first, and multiple requests are served with a single wireless broadcasting mechanism. Furthermore, to provide a balance between uploading and downloading the request, a two-step scheduling scheme is introduced, showing better performance results. In [16], Prakash et al. proposed a scheduling strategy known as a collective scheduling algorithm. The messages’ priority is achieved with three factors; the size of the message, static factor, and dynamic factor. This collective scheduling is used for clustering in VANETs. Static factors classify the safety and non-safety messages, and dynamic factors are calculated with clustering in VANETs. Based on the above three factors, the messages’ priority is calculated, and these messages are rescheduled to service and control channels. The simulation result shows that this scheme is reliable. In [17], Zhu et al. proposed architecture for Hybrid Emergency Message Transmission (HEMT) based on SDN technology on the Internet of Vehicle (IoV) in which the emergency message is transmitted to those vehicles over the area where the coverage of RSU is not entirely accessible by proposing a mechanism known as Vehicle Multi-hop Broadcast Trigger (VMBT). Through this mechanism, real-time and coverage ratio performance is improved, and the reliable transmission of emergency messages occurs in V2V communication. The simulation result shows that the scheme is scalable, reduces the controller overhead, and improves the coverage ratio’s emergency messages. There are several scheduling schemes presented in VANETs such as RSU based cloud scheduling proposed by Singh et al. in [18], declared scheduling scheme for data, voice, video, and emergency based on its weight calculated by (D∗S/W) proposed by Asgari et al. in [19]. In VANETs, the vehicle changes its position frequently due to high mobility; for this reason, Lim et al. [20] proposed a priority scheme where priority is given to high mobility vehicles based on prediction using the Markov model’s principles. In [21], Javad et al. provided a packet scheduling mechanism where priority is given based on the importance of the packets degrees by multi-level queuing. In [22], Dubey et al. proposed a scheduling policy for those in the range of RSU, and its deadline is near to expire. Moreover, it gives preference to those requests whose priority is high, but its deadline is low, due to which these messages are dropped.

## 3. Proposed Scheme

**Figure 1:**
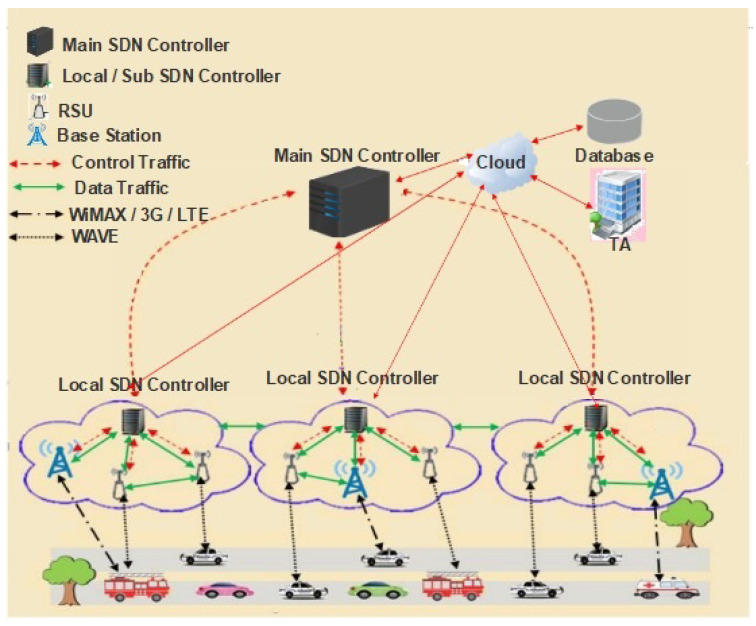
Proposed hierarchic architecture for SDVN.

In this paper, we proposed hierarchic architecture for SDVN (shown in Figure 1) contributes to the QoS in traffic management by proposing a priority-based scheduling algorithm.

To improve Intelligent Transportation Services (ITS), researchers worldwide continuously designed new architecture, routing strategies. To make VANETs systems more efficient, SDN technology is introduced with VANETs, which allows the decoupling of the data plane from the control plane, configure the network dynamically, and improved the performance. Notations used throughout this paper described in Table 1.

### 3.1. Proposed Hierarchic Architecture for SDVN

With the in-depth study of literature review and comprehensive analysis of these two networking trends (VANETs and SDN), we will move towards the SDN-based VANETs architecture. These two emerging technology (VANETs and SDN) are still under consideration and development because of its feature and real applications. Therefore, it is essential to design an efficient routing strategy for SDN-based VANETs architecture. To tackle this, we design an efficient hierarchic architecture for SDVN. The network model and proposed routing strategy are discussed below.

#### 3.1.1. Network Model

In this scheme, the network model consists of the following components as shown in the Figure 1: the main SDN controller, sub SDN controller, Base Stations (BSs), RSUs, wireless switches, and vehicles. It is a hierarchic architecture, so the network’s control plane consists of a central SDN controller at the top of its level. The lower level consists of sub SDN controllers, RSUs and BSs. The wireless switches and vehicles are present in the infrastructure layer. The following SDN components are needed for deploying the system:

#### 3.1.2. SDN Controller

The leading SDN controller builds a global view of the communication infrastructure and distributes its policy rules. Moreover, it divides the VANETs into zones of responsibility. The main SDN controller sends the global rules to each controller, which describes the network’s general behavior and has a clear scope of the entire VANETs. The SDN controllers set the rules and identify the routing parameters concerning the launch of a specific protocol. The communication between the data plane and the control plane is done on *OpenFlow* protocol. In contrast, the communication between the SDN controllers and the cloud is performed through specific APIs.

The SDN controller is a logically centralized entity in charge of (i) translating the requirements from the SDN application layer down to the SDN Datapath and (ii) providing the SDN applications with an abstract view of the network (which may include statistics and events). An SDN controller consists of one or more North Bound Interface (NBI) Agents, the SDN Control Logic, and the Control to Data-Plane Interface (CDPI) driver.

#### 3.1.3. SDN Nodes

In VANETs, nodes are vehicles equipped with On-Board Units (OBUs), making the vehicles communicate with each other by sending information directly or through Road Side Units (RSUs) deployed on the road and operating on *OpenFlow* protocol.

#### 3.1.4. SDN Road Side Unit

The RSU is a physical device that is permanently installed on the roadside. The RSU device is connected to the network to provide communication between vehicles and the SDN controller.

#### 3.1.5. Trusted Authority (TA)

The responsibility of the TA includes the registration of vehicles. It authenticates all the users registered to the VANETs environment and manages the secret parameters such as keys for all those users.

#### 3.1.6. Database

A database stores information about the network, vehicles, and their owners.

#### 3.1.7. SDN Cloud

The SDN controllers are connected to the cloud where different computations are performed, such as calculations of the car speed and distance, assessments of the road traffic situation, and perform services on a priority basis. The database is processed and managed through the cloud. The stored information in the database is updated continuously using a priority-based scheduling algorithm. The services are categorized on a priority basis for improving the QoS in VANETs.

#### 3.1.8. Priority Based Scheduling Algorithm

This section has applied the concept of a priority-based scheduling algorithm to divide messages into two categories: safety and non-safety messages. The safety messages consist of emergency messages, including hospital emergency, police helpline, rescue, natural disaster, etc. At the same time, the non-safety messages are related to user requirements such as the next traffic signal, nearest petrol pump, nearest airport, nearest shopping mall, and nearest restaurant, etc. Safety messages are the essential messages associated with human life and usually constrained by location and time (for instance, the safety information is valuable only to measure the relative distance from its original location). In this way, we can include context information with the exact time and location. The safety messages have a smaller deadline, which indicates that the data is valuable or outdated. It will be discarded if the information is outdated; otherwise, it is forwarded through the application layer for immediate response. We use an NDS method, where the message with the smallest deadline and size will be assigned first in the scheduling queue. In contrast, non-safety messages are given to the output queue on an FCFS basis.

**Figure 2:**
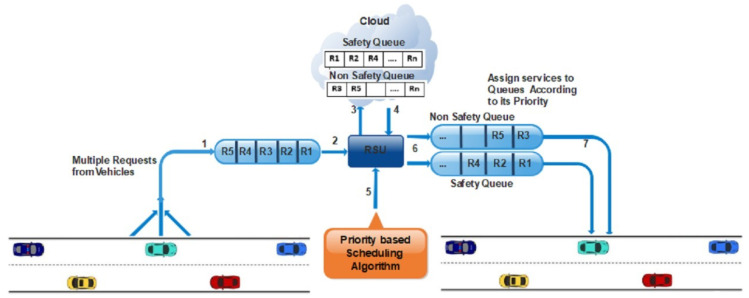
Services on priority-based scheduling.

Following are the steps for categorizing the services on priority-based scheduling as shown the above Figure 2;

1. Multiple vehicles send requests/messages for different services; these requests are stored in the queue.
2. Each request is forwarded one by one to the SDN controller.
3. The SDN controller is connected to the cloud where various computations are performed, such as services are categorized into safety and non-safety messages. Then these messages are sent back to the SDN controller.
4. Scheduling algorithm assigns the priority to emergency messages based on deadline and size. The message having the least deadline and smallest length will be considered for higher priority among all services.
5. The services are forwarded to the output queue to the given priority, as shown in Figure 2.
6. The vehicles efficiently receive their services.
7. For non-safety messages, the requests are categorized based on FCFS.

In the Algorithm 1, the vehicles send a request for different services. These requests are placed in a queue. In this case, we say List (L1) is sending to the SDN controller for further processing.
**Algorithm 1:** For Vehicles/Nodes request to cloud. **Input:** Request type **Output:** List of request L1. 1.  for (i=1;i<=n;i++)     // vehicle request i={1,2,3,⋯n} 2.   S={j1,j2,j3,⋯jn}
     // S= Request Type (vehicle can send multiple requests such as nearest ATM, nearest petrol pump, natural disaster, police helpline, rescue, etc.     // i=1⋯,n are vehicles. 3.   L1= Add request of vehicle (i) // vehicle (i)=S={S1,S2,S3⋯Sn} 4.   Return L1 5.   End of for

In the Algorithm 2, these services are categorized into safety and non-safety messages, and the two lists are prepared, i.e., List (L2) and (L3). The safety messages are placed in (L2), and the non-safety messages are placed in (L3).
**Algorithm 2:** Data categorization by cloud. **Input:** List of the request of vehicle L1 **Output:** L2 and L3 safety and non-safety list of requests. 1.   for (i=1;i<=lengthofL1;i++) 2.   if $(L1_{i}=$ (“ambulance”, “hospital emergency”, “police helpline”, “rescue” )) 3.   Assign $L1_{i}=L_{2}$ 4.   else assign $L1_{i}=L_{3}$     End if 5.   Return L2 and L3     End of for

In the Algorithm 3, the (L2) and (L3) are the lists of safety, and non-safety messages take as an input. Furthermore, for safety messages, the weight is calculated for each message based on deadline and size. Get the length and deadline of a message and then find the average length and deadline of each message, sorted in ascending order. The average difference is calculated for each message based on deadline and size. The messages that have the smallest deadline and size will be assigned first in the scheduling queue. Moreover, for non-safety messages, the priority is given based on the FCFS scheduling algorithm.
**Algorithm 3:** Prioritization of Safety and Non-Safety List (i.e., L2&L3). **Input:**L2 and L3 **Output:**
L4 and L5 lists i.e., prioritize the list of safety and non-safety messages are sent to vehicles 1.   for (i=1;i<=lengthofL2;i++)     $L4=PS_{i}=D_{i}*S_{i}$     $Q1=PS_{i}$ // Q1 is the random list of L3. 2.   for (i=1;i<=Q1.length;i++)     Find min $Q1_{i}$     $L4=\min Q1_{i}$ // Build list L4 from minimum to maximum     End for 3.   Non-Safety for (j=1; j<=length of L3; j++)     $PNS_{J}=FCFS$
     $L5=PNS_{J}$


In the Algorithm 3, the ($PS_{i}$) stands for the priority of safety messages, and ($PNS_{J}$) stands for the priority of non-safety messages.

#### 3.1.9. A Walk-trough Example

In this section, we explain the proposed model practical scenario. In VANETs, nodes are vehicles equipped with OBUs, making the vehicles communicate with each other by sending information directly or through RSUs. In real life, it is essential to provide instant communication to the vehicles for the safety of people’s precious lives. In this regard, the available model sends all the information to the center and then to the vehicles in the area’s range. Therefore, in the proposed model, we provided scheduling algorithms that differentiate between important/safety messages and non-important/non-safety messages. We can enhance the communication QoS, which means we will first prioritize urgent messages while we will send the regular messages after that. In the following example, we consider ten vehicles that send different messages for onward delivery. The messages that are sent by the vehicles are provided in the following Table 2.

In Table 2 we stored the vehicle’s requests in one RSU, the other RSUs will perform in the same way. We have ten cars from 1 to 10, and they requested eleven requests as shown in Table 2. Requests deadline and size also mentioned Table 2, as the time unit of the deadline is min and size are bytes for better understanding. Important/safety and non-safety messages will be stored in a cloud-connected with the RSUs. In the above table we have important requests are ($R_{1},R_{2},R_{4},R_{6},R_{7},R_{9}$ and $R_{10}$) and normal/non-safety requests are ($R_{3},R_{5},R_{8},R_{11}$). After applying our scheduling algorithms, the following Table 3 and Table 4 will be generated accordingly, as we first will use priority basis scheduling algorithm and then FCFS algorithm.

In the proposed priority basis scheduling algorithm, each message’s weight is calculated based on deadline and size. Get the length and deadline of a message and then find the average length and deadline of each message, sorted in ascending order. The average difference is calculated for each message based on deadline and size. The messages/request that has the smallest deadline and size will then be sent first, then the other messages. Table 3 our proposed system generated the seven important messages deadline and size and made them in ascending order for further sending. $R_{9}$ has a short deadline and size so that it will be sent first, then $R_{1}$ will be sent, and so on as provided in Table 3.

Table 4 is the output of FCFS algorithm, the non-important messages requests ($R_{3}$, $R_{5}$, $R_{8}$, $R_{11}$) will be sent after important messages. Therefore, in this way, our proposed system will work and will communicate the messages of vehicles. After communication, the data will be stored in the cloud for future use.

**Figure 3:**
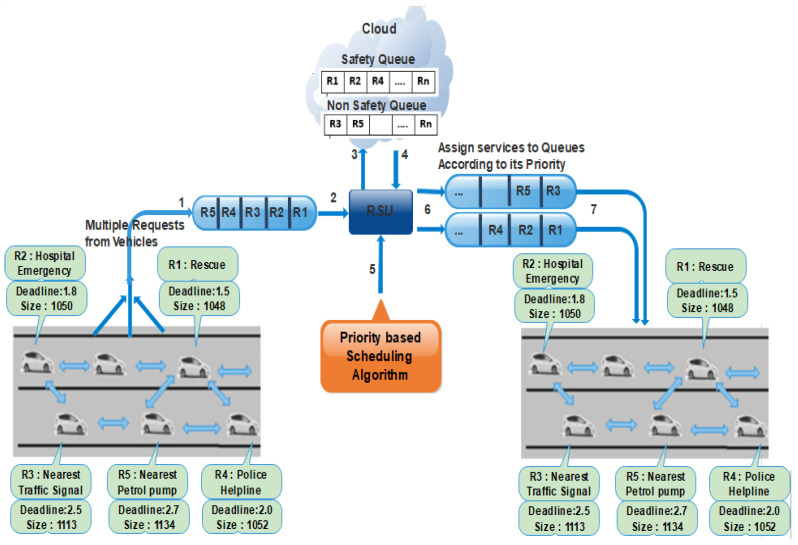
A walk through Example of PSA.

In VANETs, fast and QoS-based communication is required; for instance, if one node wants to send messages, which is a common conversation (just asking for the nearest petrol pump, nearest restaurant, nearest bank, etc.) and one another node is passing some important information such as informing the other vehicles about Fog, accident, thieves or asking helping for hospital emergency, police helpline and rescue, etc. In this case, we need to send the important messages and then move on to the other information. Here, we need such a mechanism/algorithm that decides which message needs to be sent first and then the other messages. We enhance the entire VANETs communication process, and we categorize traffic flow messages into safety and non-safety messages. The safety messages consist of emergency messages, including hospital emergency, police helpline, rescue, natural disaster, etc. At the same time, the non-safety messages are related to user requirements such as the next traffic signal, nearest petrol pump, nearest airport, nearest shopping mall, and nearest restaurant, etc. After categorization of safety and non-safety messages, we will give priority to safety messages. Because the safety message is associated with the life of human and usually constrained by location and time (for instance, the safety information is valuable only to measure the relative distance from its original location) so in this way, we can include the context information with exact time and location as (for example, accident-Friday 11 am -Location: MN- [X, Y]) in Information Object (IO) name. The safety content has a smaller deadline, and from this context information, it is checked that the information is valuable or outdated. If the information is outdated, then it will be discarded. Otherwise, it is forwarded through the application layer. For this reason, we used a priority-based scheduling algorithm for safety messages using the NDS method. In this method, the message having the least deadline and smallest length will be considered for higher priority among all services as shown in Figure 3. For safety messages, the weight is calculated for each message based on deadline and size. Get the length and deadline of a message and then find the average length and deadline of each message, sorted in ascending order. The average difference is calculated for each message based on deadline and size. The messages that have the smallest deadline and size will be assigned first in the scheduling queue. For non-safety messages, the requests are categorized based on FCFS. In this way, the vehicles efficiently receive their services within the proper time.

## 4. Simulation and Analysis of Priority-Based Scheduling Algorithms

In this section, we present the proposed model simulation setup and evaluation of the model. We use the *CloudSim* toolkit to simulate the proposed priority-based scheduling algorithms.

### 4.1. Simulation Setup

The *CloudSim* [23] toolkit has been used to simulate the proposed priority-based scheduling algorithm. This framework is used for modeling and simulation of cloud computing services. There are two types of scheduling queues, such as safety and non-safety. In the safety queue, every message is scheduled based on length and deadline. The message that has the smallest deadline and size will be assigned first in the scheduling queue. For the non-safety queue, the messages are processed based on the FCFS method.

### 4.2. Experimental Evaluation

We created a data center, having a processing rate is 1000 Million Instructions Per Second (MIPS), and memory is 512 MB. Table 5 consists of a detailed description of the data center configuration, and Table 6 describes the configuration of the simulated cloud. In the first step, we got the length and deadline of a cloudlet and then found the average length and deadline of each cloudlet, which are sorted in ascending order in the lists. The average difference is calculated for each cloudlet based on deadline and size, and the cloudlets that have the smallest deadline and size are assigned first in the scheduling queue. A Cloudlet is a representation of a task in *CloudSim*. Cloudlet is defined, in *CloudSim*, as a job submitted to the cloud. In this case, jobs are the messages that are assigned to the cloud. For non-safety messages, the priority is given based on the FCFS scheduling algorithm.

### 4.3. Simulation Result

**Figure 4:**
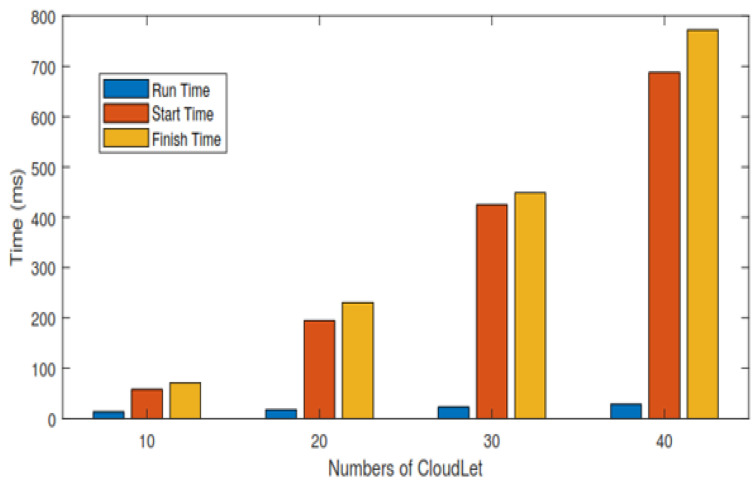
The life cycle of safety messages.

**Figure 5:**
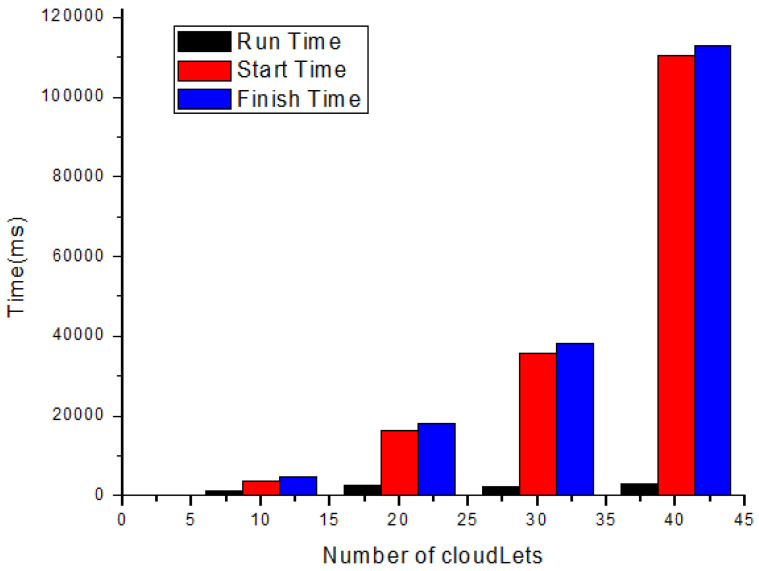
Life Cycle of non-safety messages.

**Figure 6:**
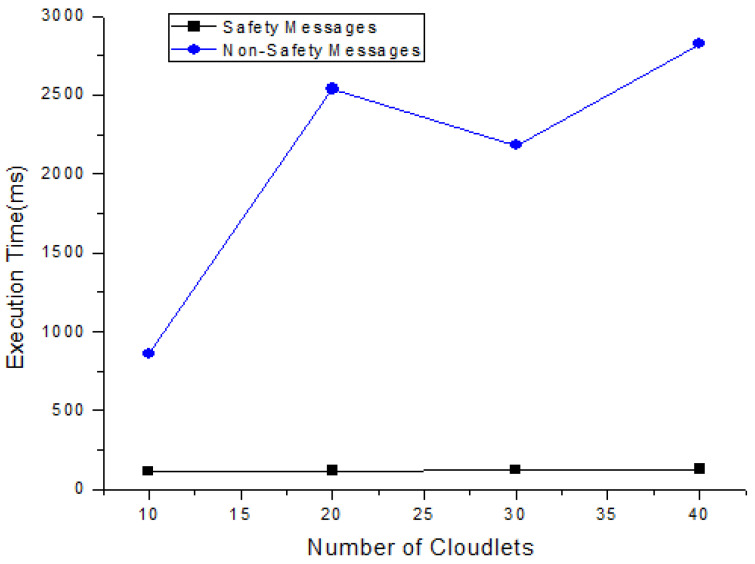
Comparison of calculated execution time for safety messages and non-safety messages.

In this section, each task’s total execution time is calculated in the cloud by adopting the scheduling policy based on deadline and size. Figure 4 and Table 7 show the expected calculated execution time for safety messages based on the sum of the start and running time. Figure 5 and Table 8 show the expected calculated execution time for non-safety messages. Figure 6 shows the comparison of safety and non-safety messages in terms of the computed execution time, which shows better results than non-safety messages.

**Figure 7:**
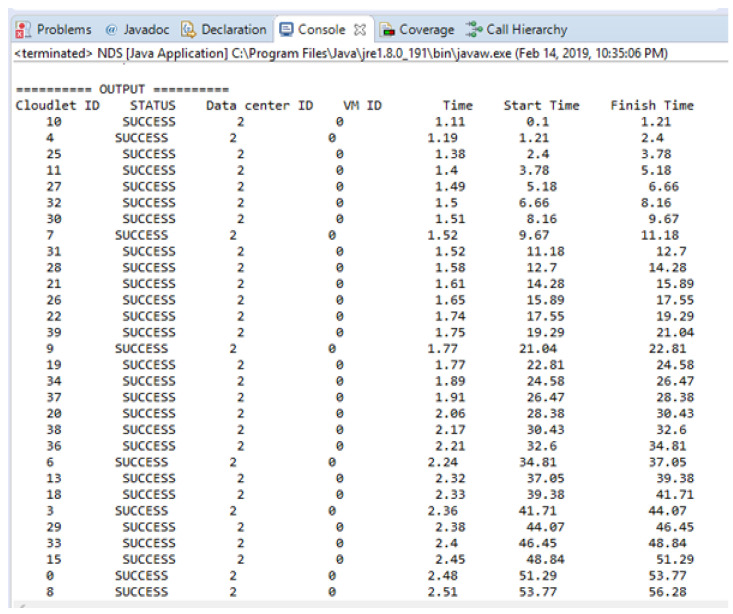
Experimental results in term of execution time for scheduling safety messages.

Figure 7 shows the experimental result in terms of execution time for scheduling safety messages based on NDS. Figure 7 is shown the simulation results run on the cloudlets that are successfully executed by the datacenters. The time unit here in this Figure 7 is ms.

Table 7, we calculate the result of 10 cloudlets based on the sum of start and running time, and the average result is calculated for 10 cloudlets and then 20, 30, and 40 cloudlets as well for safety messages. The time unit of Table 7 in ms.

Figure 4 shows the expected calculated execution time for safety messages based on the sum of start and running time for 10, 20, 30, and 40 cloudlets.

**Figure 8:**
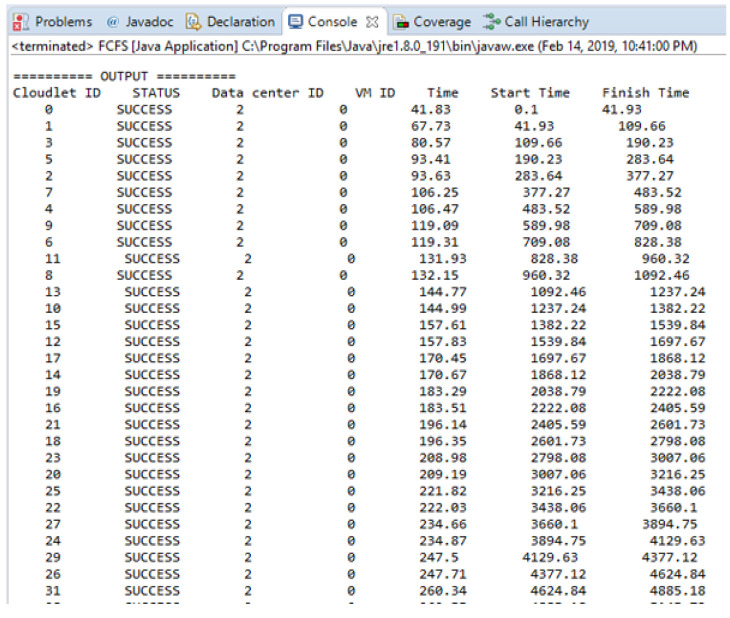
Experimental result in term of execution time for scheduling non safety messages.

In this section, the experimental result is carried out for 40 messages, and the execution time of each task is calculated in the cloud by adopting an FCFS basis. Figure 8 shows the experimental result in term of execution time for scheduling non-safety messages based on FCFS. Figure 8 is shown the simulation results run on the cloudlets that are successfully executed by the datacenters. The time unit here in this Figure 8 is ms.

Table 8 we calculate the result of 10 cloudlets based on the sum of start and running time, and the average result is calculated for 10 cloudlets and then 20, 30, and 40 cloudlets as well for non-safety messages. The time unit of Table 8 is ms.

Figure 5 shows the expected calculated execution time for non-safety messages based on the sum of start and running time for 10, 20, 30, and 40 cloudlets.

The above Figure 6 shows the comparison of calculated execution time for safety messages and non-safety messages. The calculated execution time of 40 messages is carried out for safety and non-safety messages and we see that the safety messages are executed in less time as compared to non-safety messages.

## 5. Conclusions

QoS is the main research concerns in designing our proposed SDVN architecture. QoS in traffic management is achieved by a Priority-based Scheduling Algorithm (PSA), where messages are categorized into two queues, i.e., safety queue and non-safety queue. In the safety queue, the messages are prioritized based on deadline and size using NDS as the safety messages are human life critical and constrained by location and deadline. In contrast, the non-safety queue is prioritized based on the FCFS method. We used the CloudSim toolkit to simulate the proposed PSA. The experimental result is carried out for 40 messages, and the execution time of each task is calculated in the cloud by adopting an NDS method for safety messages and an FCFS method for non-safety messages. We also calculated the result of 10 cloudlets based on the sum of start and running time, and the average result is calculated for 10 cloudlets and then 20, 30, and 40 cloudlets as well for safety messages and non-safety messages. Finally, the comparison of safety and non-safety messages in terms of the computed execution time and the PSA simulation result shows better results than non-safety messages in terms of execution time.

Future Direction- In the future, we are going to design a novel and efficient cryptosystem based on PKI-based digital signature for secure communication between Vehicle to Vehicle (V2V), public key authority infrastructure for Vehicle to Infrastructure (V2I), and a three-way handshake mechanism for the secure communication between main and sub-SDN controllers. The security validity of the proposed scheme will be check using a new familiar simulation tool called AVISPA.

## Figures and Tables

**Table 1:** Notations and Description.

Notations	Description
AU	Application Unit
QoS	Quality of Service
SDN	Software Defined Networks
VANETs	Vehicular Adhoc Networks
PSA	Priority-based Scheduling Algorithm
NDS	New Deadline and Size of data method
FCFS	First Come First Serve
SDVN	Software Defined Vehicular Network
ITS	Intelligent Transportation System
MANETs	Mobile Ad hoc Networks
V2V	Vehicle-to- Vehicle
V2R	Vehicles-to-Roadside
V2I	Vehicles-to-Infrastructure
API	Application Programming Interface
MEC	Multi-access Edge Computing
SVAO	SDN Based Vehicle Ad hoc On-Demand
OLSR	Optimize Link State Routing
DSR	Dynamic Source Routing
DSDV	Destination Sequence Distance Vector
DB	Distance Based routing
UAV	Unmanned Aerial Vehicle
p−RSA	priority-based RSA algorithm
SDF	Shortest job based on Data First
HEMT	Hybrid Emergency Message Transmission
IoV	Internet of Vehicle
RSU	Road Side Unit
VMBT	Vehicle Multi-hop Broadcast Trigger
BSs	Base Stations
NBI	North Bound Interface
SBI	South Bound Interface
OBUs	On-Board Units
TA	Trusted Authority
$PS_{i}$	Priority of Safety Messages
PNSJ	Priority of Non Safety Messages
MIPS	Million Instructions Per Second
SDNE	Software Defined Network Edge
RTISAR	Real-Time Intersection-Based Segment Aware Routing
MEC	Multi-access Edge Computing

**Table 2:** Ten vehicles messages that want to send.

S. No.	Vehicle Name	Message/Service Requests ($R_{n}$)	Size of Message (Bytes)	Deadline of Message (min)
1	Car 1	Car 1_$R_{1}$ = “Rescue help”	1048	1.5
2	Car 2	Car 2_$R_{2}$ = “Hospital Emergency”	1050	1.8
3	Car 3	Car 3_$R_{3}$ = “Nearest traffic signal”	1113	2.5
4	Car 4	Car 4_$R_{4}$ = “Police help, suspicious movement”	1052	2
5	Car 5	Car 5_$R_{5}$ = “Nearest petrol help”	1134	2.7
6	Car 6	Car 6_$R_{6}$ = “Rescue help, accident”	1030	2.8
7	Car 7	Car 7_$R_{7}$ = “Hospital Emergency, High BP”	1300	3.1
8	Car 8	Car 8_$R_{8}$ = “Nearest traffic signal” Car 8_$R_{9}$ = “Police help, thieves”	1202 1208	1.1 1
9	Car 9	Car 9_$R_{10}$ = “Police help, informing about road robbery”	1133	2
10	Car 10	Car 10_$R_{11}$ = “Nearest petrol help”	1070	3

**Table 3:** Priority basis scheduling algorithm output in ascending order.

S. No.	Service Requests ($R_{n}$)	Deadline (m) & Size (Bytes)	Remarks
1	$R_{9}$	1208	$R_{9}$ will be send first
2	$R_{1}$	1572	$R_{1}$ will be send second
3	$R_{2}$	1890	$R_{2}$ will be send third
4	$R_{4}$	2104	$R_{4}$ will be send fourth
5	$R_{10}$	2266	$R_{10}$ will be send fifth
6	$R_{6}$	2884	$R_{6}$ will be send six
7	$R_{7}$	4030	$R_{7}$ will be send seven

**Table 4:** FCFS basis scheduling algorithm output.

S. No.	Message/Service Requests ($R_{n}$)	Remarks
1	$R_{3}$	$R_{3}$ arrives first so it will be sent first
2	$R_{5}$	$R_{5}$ arrives second so it will be sent second
3	$R_{8}$	$R_{8}$ arrives third so it will be sent third
4	$R_{11}$	$R_{11}$ arrives fourth so it be sent fourth

**Table 5:** Configuration of Simulated Cloud.

Cloud	Number
No. of Datacenter	1
No. of Cloudlet	40
No. of Broker	1
No. of Virtual Machines	1

**Table 6:** Configuration of Data Center.

Data Center	Configuration
Architecture	x86
RAM (MB)	512
Hypervisor	Xen
Storage (MB)	10,000
MIPS	1000
Bandwidth (MBps)	1000

**Table 7:** Total calculated execution time for safety messages.

No. of Cloudlets	Run Time (ms)	Start Time (ms)	Finish Time (ms)
10	960.22	3613.79	4574.01
20	2641.34	16,444.33	18,085.74
30	2283.45	35,747.62	38,031.07
40	2925.7	110,361.11	112,962.04

**Table 8:** Total calculated execution time for non safety messages.

No. of Cloudlets	Run Time (ms)	Start Time (ms)	Finish Time (ms)
10	13.47	58.06	71.45
20	17.729	194.38	230.79
30	22.9	424.49	448.248
40	28.53	687.26	771.99s

## Data Availability

Not applicable.
